# Thyroid hormone suppresses expression of stathmin and associated tumor growth in hepatocellular carcinoma

**DOI:** 10.1038/srep38756

**Published:** 2016-12-09

**Authors:** Yi-Hsin Tseng, Ya-Hui Huang, Tzu-Kang Lin, Sheng-Ming Wu, Hsiang-Cheng Chi, Chung-Ying Tsai, Ming-Ming Tsai, Yang-Hsiang Lin, Wei-Chun Chang, Ya-Ting Chang, Wei-Jan Chen, Kwang-Huei Lin

**Affiliations:** 1 Graduate Institute of Biomedical Sciences, College of Medicine, Chang Gung University, Taoyuan 333, Taiwan, Republic of China; 2 Liver Research Center, Chang Gung Memorial Hospital, Linko, Taoyuan 333, Taiwan, Republic of China; 3 Division of Neurosurgery, Chang Gung Memorial Hospital-Linkou & Chang Gung University, Taoyuan 333, Taiwan, Republic of China; 4 Department of Nursing, Chang-Gung University of Science and Technology, Taoyuan 333, Taiwan, Republic of China; 5 Molecular Medicine Research Center, Chang Gung University, Taoyuan 333, Taiwan, Republic of China; 6 First Cardiovascular Division, Chang Gung Memorial Hospital, Taoyuan 333, Taiwan, Republic of China

## Abstract

Stathmin (STMN1), a recognized oncoprotein upregulated in various solid tumors, promotes microtubule disassembly and modulates tumor growth and migration activity. However, the mechanisms underlying the genetic regulation of STMN1 have yet to be elucidated. In the current study, we report that thyroid hormone receptor (THR) expression is negatively correlated with STMN1 expression in a subset of clinical hepatocellular carcinoma (HCC) specimens. We further identified the *STMN1* gene as a target of thyroid hormone (T_3_) in the HepG2 hepatoma cell line. An analysis of STMN1 expression profile and mechanism of transcriptional regulation revealed that T_3_ significantly suppressed STMN1 mRNA and protein expression, and further showed that THR directly targeted the *STMN1* upstream element to regulate *STMN1* transcriptional activity. Specific knockdown of STMN1 suppressed cell proliferation and xenograft tumor growth in mice. In addition, T_3_ regulation of cell growth arrest and cell cycle distribution were attenuated by overexpression of STMN1. Our results suggest that the oncogene *STMN1* is transcriptionally downregulated by T_3_ in the liver. This T_3_-mediated suppression of STMN1 supports the theory that T_3_ plays an inhibitory role in HCC tumor growth, and suggests that the lack of normal THR function leads to elevated STMN1 expression and malignant growth.

Thyroxine, also known as 3,3′,5-triiodo-L-thyronine (T_3_), mediates numerous physiological processes, including ontogenesis, cell growth, cellular differentiation and metabolism, in nearly all mammalian tissues. The biological activity of T_3_ relies on binding to nuclear thyroid hormone receptors (THRs) belonging to the ligand-dependent transcriptional factor family, which maintain homeostasis by modulating expression levels of various genes. Two human THR genes, TRα (*THRA*) and TRβ (*THRB*), are located on human chromosomes 17 and 3, respectively^1^. Different isoforms of THR (TRα1, TRα2/TRβ1 and TRβ2) are generated by alternative RNA splicing or multiple promoter usage^2^. Moreover, THRs usually interact with retinoid X receptor (RXR) to form heterodimers that bind to thyroid hormone response elements (TREs) within the promoter regions or introns of target genes to regulate their transcriptional activity^3^.

Disorders of the thyroid gland are increasingly common endocrine diseases^4^. The lack of T_3_ causes goiter and metabolic syndromes, such as mental retardation^5^. The liver expresses equal amounts of THRA and THRB, implying that T_3_ regulates gene expression through transactivation^6^. To date, several studies have confirmed that hypothyroidism triggers hyperlipidemia, obesity and non-alcoholic steatohepatitis, the latter of which progresses to liver cirrhosis and hepatocellular carcinoma (HCC) development^7^,^8^. A significantly increased risk of HCC development (up to 2–3 fold) has been reported in human adults with hypothyroidism^9^. Moreover, studies on patients with chronic hepatitis C virus infection have suggested a correlation between lower T_3_ levels and thyroid papillary cancer^10^,^11^. Notably, chemical-induced liver cancer in rats was shown to be markedly reduced in the presence of T_3_^12^. These findings suggest a significant association of T_3_ malfunction and impaired liver function with the pathogenesis of cancer.

Analogously, aberrant THR expression or mutations have been reported in cases of severe resistance to thyroid hormone and are associated with developmental disease and cancer progression. Genetic mutations in THRA and THRB were detected in 65% and 76% of HCCs, respectively^13^. A characterization of mutant THRs in the J7 human hepatocellular carcinoma cell line revealed that mutated THRA binds TREs, but not T_3_, indicative of dominant-negative activity^14^,^15^. THRs play an important role in tumor progression, as evidenced by their aberrant expression and mutation in other human cancers, including pituitary tumors, thyroid papillary cancer and renal clear-cell carcinomas^16^,^17^,^18^,^19^. Transgenic mice harboring a THRB mutation (THRB^pv/pv^) isolated from patients with thyroid hormone resistance exhibit spontaneous induction of metastatic thyroid carcinomas^20^. Loss of functional THRs in mice leads to the development of follicular thyroid cancer and metastases in the lung^21^. Moreover, THRB overexpression potently represses tumor metastasis^22^. These findings collectively suggest that loss of normal regulation of THRs enhances tumor progression, supporting a tumor-suppressor function of these receptors. Conversely, however, other studies have indicated that THRs enhance tumor progression. For instance, T_3_ has been reported to stimulate the proliferation of various cancer cell types, including pituitary-derived cancer, breast cancer, prostate cancer, and glioma^23^,^24^,^25^,^26^. Previous experiments by our group showed that T_3_ suppresses hepatoma cell growth by prolonging the G0/G1 phase while inducing cell migration in association with enhanced matrix metallopeptidase (MMP) activity^27^,^28^. Thus, the complex roles of T_3_/THR in tumorigenesis appear to reflect distinct, tissue-specific genetic backgrounds and definitions of oncogenic roles. The details of the regulatory mechanisms involved in these oncogenic processes remain to be established.

Stathmin (STMN1, also known as oncoprotein 18 [OP18]) is a 149-amino-acid, cytosolic protein that is highly conserved among vertebrates^29^,^30^. STMN1 is highly expressed in various cancers and has been characterized as an oncogenic protein^31^. The predominant molecular function of STMN1 is regulation of microtubule dynamics. The phosphorylated C-terminal domain of STMN1 physically interacts with unpolymerized tubulin dimers, influencing the dynamics of microtubule formation^32^,^33^. STMN1 prevents assembly and promotes disassembly of microtubules, thus participating in microtubule-related cellular functions, such as cell proliferation and migration. STMN1 is downregulated by p53 and regulates cell cycle arrest at the G2/M and G1/S checkpoints^34^,^35^. Moreover, STMN1 interacts with p27 and Cdk2/Cdk5, leading to enhanced protein phosphorylation and consequent tubulin stabilization and inhibition of cell migration^36^. These findings support a crucial role of STMN1 in cancer growth and mobility.

Accumulating evidence suggests that STMN1 regulates microtubulin assembly and consequently tumorigenesis, but the mechanisms underlying the regulation of *STMN1* gene expression remain unknown. Here, we found that THR and STMN1 are negatively correlated at RNA and protein levels in clinical specimens. We further determined that STMN1 expression was markedly repressed by T_3_ at the transcriptional level. Moreover, cell growth was inhibited by STMN1 depletion as well as T_3_ treatment, confirming that T_3_ plays a role in suppression of tumor growth.

## Results

### Negative correlation of THRA and STMN1 expression in clinical liver cancer specimens

**Figure 1:**
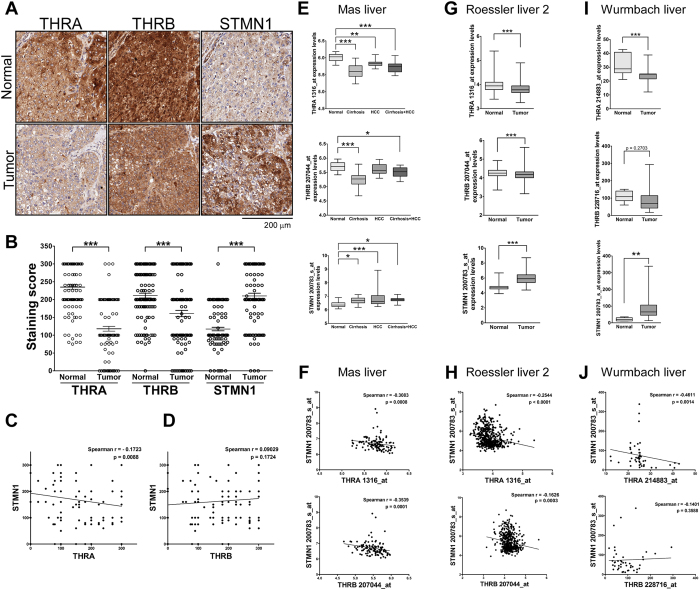
Negative correlation of thyroid hormone receptor and STMN1 expression levels in clinical HCCs. (**A**) Immunohistochemical staining of THR and STMN1 in normal livers (N = 115) and HCCs (N = 115). Staining score were compared and plotted in (**B**). (**C**,**D**) Correlations of THRs and STMN1 expression levels were analyzed and indicated. (THRA vs. STMN1, Spearman r = −0.1723; 95% CI, −0.2986 to −0.04003; p = 0.0088. THRB vs. STMN1, Spearman r = 0.09029; 95% CI, −0.04341 to 0.2208; p = 0.1724). (**E–J**) THRA, THRB and STMN1 mRNA expression levels in three public liver cancer datasets from Oncomine were analyzed. (**E**,**F** Mas Liver, N = 115; **G**,**H** Roessler liver 2, N = 488; **I,J** Wurmbach liver, N = 75, respectively) (***P < 0.001; **P < 0.01, *P < 0.05). Error bars, s.e.m.

Previous studies have reported THR-mediated suppression of cell proliferation^28^ and loss of THR expression in clinical samples of HCC^37^,^38^. In contrast, several lines of evidence indicate that STMN1 is upregulated in cancers^31^. To gain insight into the biological significance of these expression patterns, we compared the relative abundance of THR isoforms (THRA, THRB) and STMN1 proteins in clinical HCC specimens (Fig. 1A). Immunohistochemical findings revealed that expression of THRs was markedly decreased in tumor specimens, whereas STMN1 levels were enhanced (Fig. 1B). Moreover, expression of THRA, but not THRB, was negatively correlated with that of STMN1 in patients (Fig. 1C and D). To further clarify the negative correlation between specific THR isoforms and STMN1, we analyzed three public datasets from Oncomine (Fig. 1E–J). Notably, THRA and THRB mRNA expression were decreased, whereas expression of STMN1 was enhanced, in tumor specimens (Fig. 1E,G and I). Importantly, the negative correlation between STMN1 and THRA was stronger than that between STMN1 and THRB (Fig. 1F,H and J).

### T_3_ suppresses STMN1 expression in HepG2 cell lines

**Figure 2:**
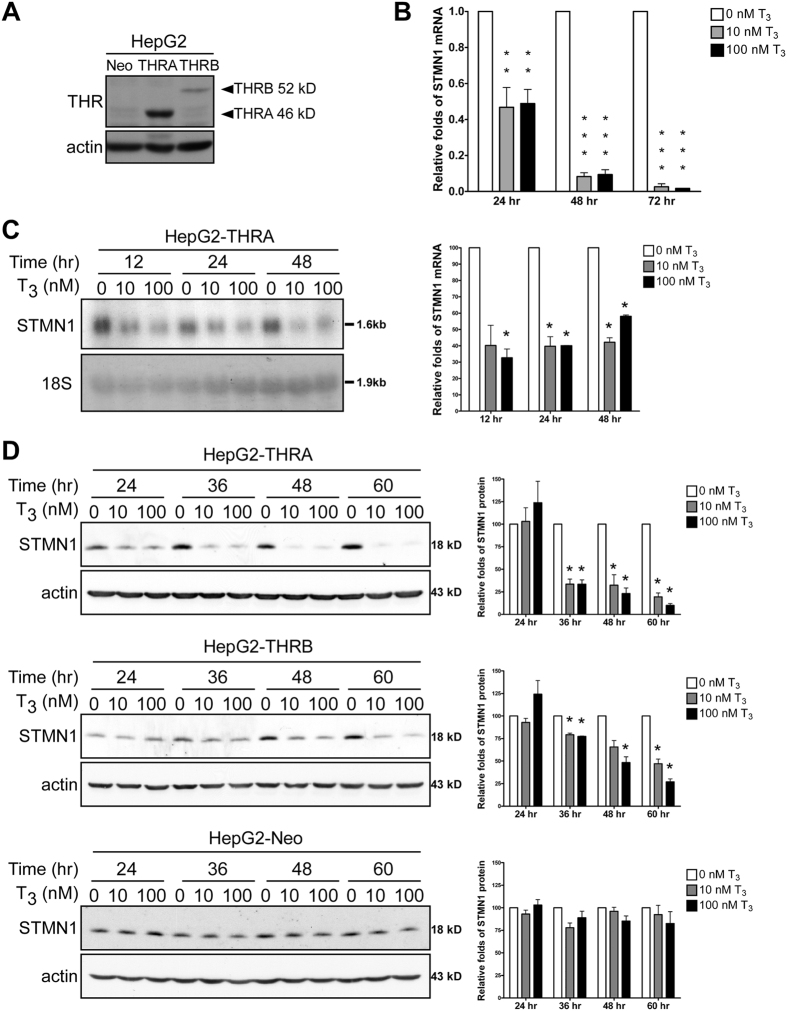
T_3_ represses STMN1 expression in HepG2 cell lines. (**A**) THR protein expression levels in HepG2 control and THR overexpressing cell lines. (**B**) qRT-PCR of STMN1 mRNA expression levels in HepG2-THRA cells (**P < 0.01, ***P < 0.001, n = 3). (**C**) Northern blots of HepG2-THRA cell RNAs. (*P < 0.03, n = 2). (**D**) Immunoblots of HepG2-THRA, HepG2-THRB and HepG2-Neo cell lysates (*P < 0.03, n = 3). Error bars, s.e.m. Electrophoretic gels and blots in each experiment were conducted under the same experimental conditions, and images were cropped.

Next, we overexpressed THRA in HepG2 cells, which express low levels of endogenous THRs (Fig. 2A and S1A). We then verified suppression of STMN1 mRNA expression by T_3_ in THRA-overexpressing HepG2 cells using quantitative reverse transcription-polymerase chain reaction (qRT-PCR) (Fig. 2B, S1B and S1C). After 72 h in the presence of T_3_, STMN1 mRNA levels were less than 10% of control levels (fold-repression values were normalized to those in the absence of T_3_ at each time point). The effects of T_3_ on endogenous STMN1 mRNA levels in THRA-overexpressing cells were further analyzed by Northern blotting, which confirmed that STMN1 mRNA levels were repressed by T_3_ (Fig. 2C, right panel). Of note, qRT-PCR analysis shows higher sensitivity that STMN1 mRNA levels have been reduced to 20% in 48 h. Next, we examined whether T_3_ exerts an inhibitory effect on STMN1 protein expression. Immunoblot analyses revealed that STMN1 protein was markedly suppressed by T_3_ in cells ectopically expressing THR compared with that in cells devoid of THR (Fig. 2D; -THRA, -THRB and empty vector-Neo, Supplementary Fig. S1D and E; -THRA and empty vector-Neo, respectively). These data collectively demonstrate that T_3_ suppresses STMN1 mRNA and protein expression in a concentration- and THR-dependent manner.

### T_3_ represses STMN1 expression at the transcriptional level

**Figure 3:**
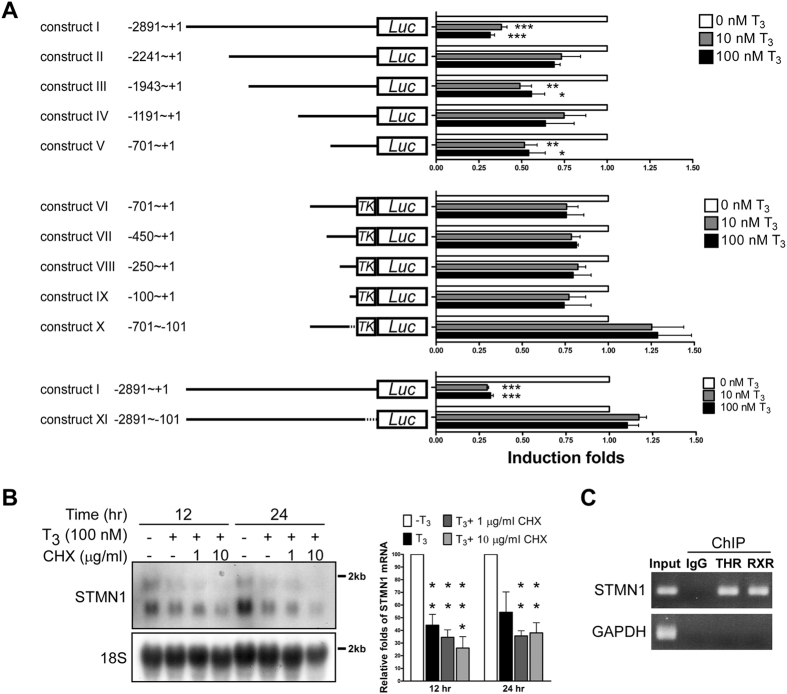
Thyroid hormone receptor represses STMN1 in HepG2 cell at the transcription level directly. (**A**) Schematic representation of the STMN1 promoter (+1, translation start site). A serial deletion fragments of STMN1 5′-flanking DNA were cloned into pGL3 (construct I~V, XI) or pGL3tk-luc (construct VI~X) reporter plasmids, as indicated. The T_3_ repression folds were presented as mean values ± s.e.m. (n = 3). (**B**) HepG2-THRA was incubated for 12–24 h with 0 to 10 μg/ml cycloheximide (CHX) in the absence or presence of T_3_, after which total RNA was isolated and sequentially analyzed by Northern blot. (n = 3, Error bars, s.e.m.). (**C**) ChIP assay of STMN1 5′-flanking region (−101~+1). The promoter region of GAPDH was used as the negative control. Electrophoretic gels and blots in each experiment were conducted under the same experimental conditions, and images were cropped.

Next, we determined whether the repression of *STMN1* gene expression by T_3_ is attributable to THR-mediated transcriptional regulation. To this end, we created a promoter-luciferase reporter construct by cloning a 3-kb genome sequence upstream of the *STMN1* start codon into a pGL3-Luc vector and performed luciferase assays. As shown in Fig. 3A, the 3-kb upstream region (construct I, positions −2891 to +1) was involved in mediating T_3_-induced repressive activity (Fig. 3A, upper panel). A serial deletion analysis of the *STMN1* promoter further revealed that the −701 to +1 fragment contains the element responsible for T_3_ suppression of *STMN1* transcription (Fig. 3A, construct V). Further deletion impaired *STMN1* promoter activity, suggesting that these additionally deleted regions might be responsible for native RNA polymerase II recognition (Supplementary Fig. S2). Accordingly, the −701 to +1 region and serially truncated fragments were subcloned into a pA3tk-Luc vector containing a minimum thymidine kinase promoter to provide basal transcriptional activity (Fig. 3A, constructs VI–X). The transcriptional activity of −100 to +1 as well as −701 to +1 regions was suppressed by T_3_ (constructs VI and IX, respectively). Furthermore, T_3_ suppression of transcriptional activity was blunted in the absence of the −100 to +1 fragment (Fig. 3A, construct X, −701 to −101, and construct XI, −2891 to −101). Collectively, these data indicate that a suppressive TRE is present upstream of the *STMN1* start codon and mediates negative regulation of *STMN1* transcription by T_3_.

Since THRs act as nuclear transcription regulators, we examined whether THR directly targets the *STMN1* promoter to downregulate *STMN1* transcription. Experiments using the protein synthesis inhibitor, cycloheximide (CHX), showed that STMN1 mRNA levels remained suppressed upon T_3_ treatment in the absence of *de novo* protein synthesis (Fig. 3B). Quantification of these results revealed that co-treatment with CHX suppressed STMN1 mRNA levels to an extent similar to that of T_3_ treatment alone (Fig. 3B, right panel). These data imply that THR regulates the *STMN1* promoter directly and not through an intermediate transcription factor. If this were not the case, the intermediate transcription factor protein should be inhibited by CHX, resulting in impaired T_3_ repression of *STMN1* expression. To validate specific THR binding to the STMN1 genome, we performed chromatin immunoprecipitation (ChIP) assays. These assays revealed a THR-associated signal in the upstream element (positions −101 to +1) of *STMN1* (Fig. 3C, lane 3; compare to IgG control in lane 2). This fragment was additionally pulled down by RXR, which dimerizes with THR (Fig. 3C, lane 4), suggesting that THR and RXR co-bind this sequence to exert repressive activity. On the basis of these findings, we suggest that THR physically binds this upstream element, leading to suppression of *STMN1* transcription by T_3_. Furthermore, in view of previous and present results, we speculate that STMN1 is negatively regulated by T_3_; thus, its expression levels are elevated in THR-deficient HCCs.

### Depletion of STMN1 in J7 cells suppresses cell proliferation

**Figure 4:**
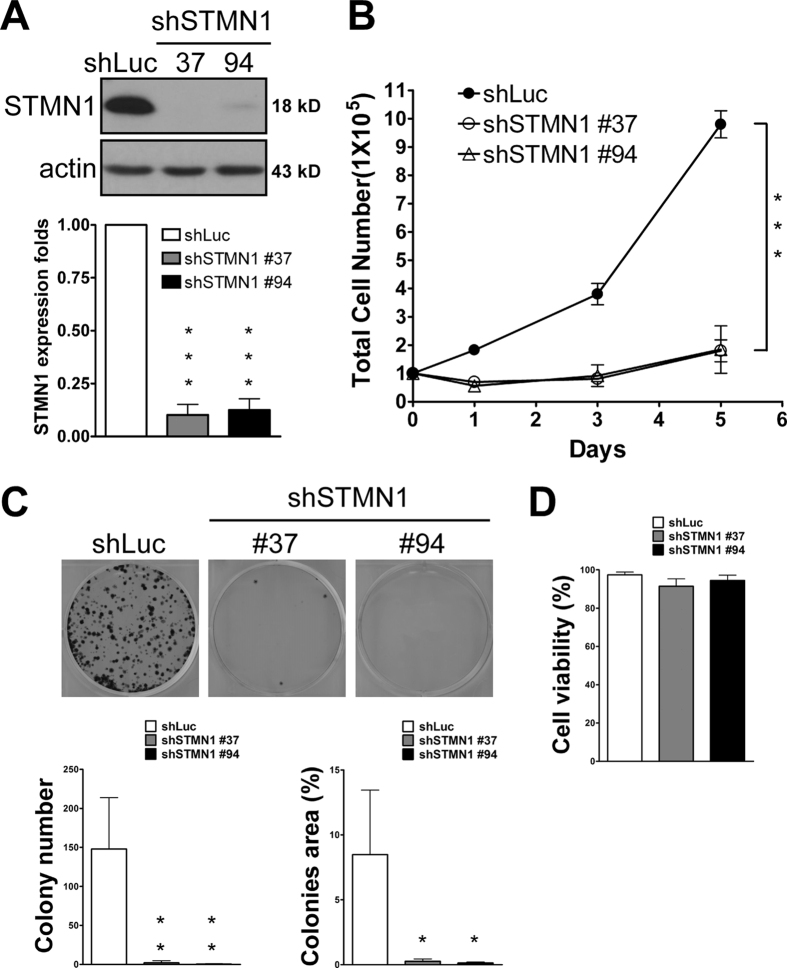
Knockdown of STMN1 in J7 represses cell proliferation. (**A**) Immunoblots of J7 knockdown STMN1 (shSTMN1) and cognate control (shLuc) cells. Repressed protein levels were indicated. (**B**) For proliferation assay, 1*10^5^ cells were seeded in 6-well plate. At the indicated day, cells were trypsinized and counted. (**C**) For colony formation assay, 2*10^3^ cells were seeded in 6-well plate for 14 days. (**D**) Trypan blue staining of viable cell was examined. (***P < 0.001; **P < 0.01; *P < 0.05 n = 3). Error bars, s.e.m. Gel electrophoresis was conducted under the same experimental conditions, and images of blots were cropped.

We further examined the effects of STMN1 knockdown on cell growth. Because HepG2 cells are inadequate for tumor xenograft models, we chose J7 cells for proliferation assays and xenograft transplantation. Silencing of STMN1 in J7 cells by transduction with a lentivirus expressing small hairpin RNA (shRNA; clones #37 and #94) against STIMN1 led to marked suppression of STMN1 expression and growth of J7 liver cells compared with cells transduced with control lentivirus shRNA targeting firefly luciferase (Fig. 4A and B). Only a few colonies were detected in colony-formation assays (Fig. 4C). In contrast, cell viability was not significantly reduced by knockdown of STMN1 (Fig. 4D).

**Figure 5:**
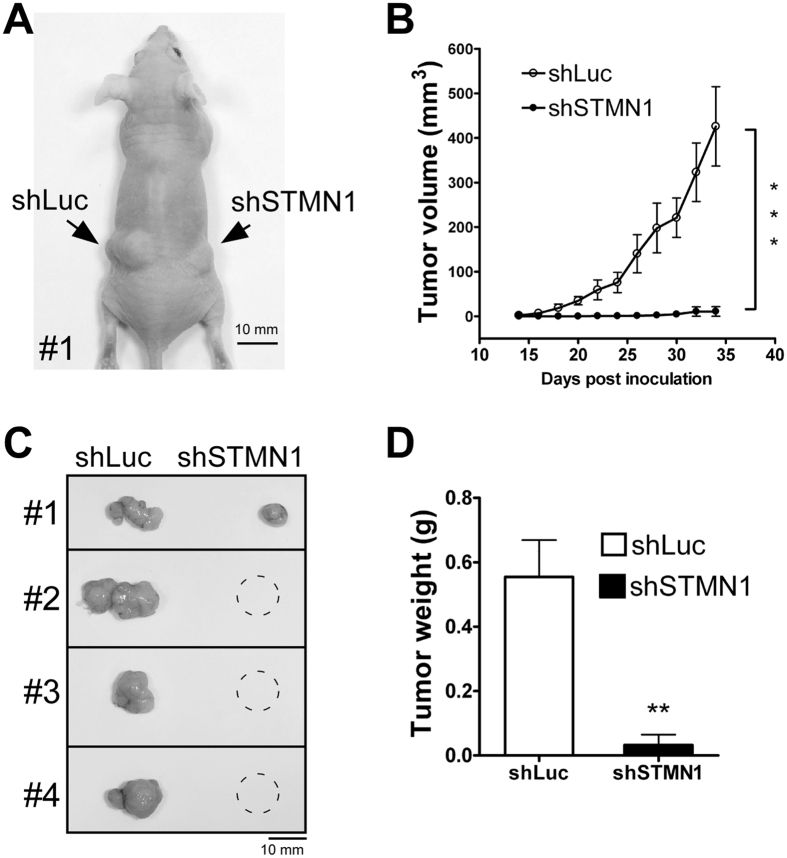
Knockdown of STMN1 in J7 represses xenograft tumor growth. (**A**) Subcutaneous injection of J7-shSTMN1 cells in nude mice. J7-shSTMN1 was generated from the pooled lentivirus (shSTMN1#37 and shSTMN1#94, 1:1). Briefly, 1*10^6^ cells were suspended in 150 μl of PBS and injected into dorsal skin of mice. Two weeks later, visible tumor size was measured for 3 weeks (**B**). The mice were finally sacrificed at 5 weeks, and xenograft tumors were dissected (**C**) and weighed (**D**) (***P < 0.001; **P < 0.01 n = 4). Error bars, s.e.m.

These findings indicate that STMN1 depletion significantly reduces cell growth. To confirm this phenomenon, we established a heterotopic xenograft model to test tumorigenesis potential *in vivo*. STMN1-knockdown cells and parental control cells were transplanted in parallel in the dorsal skin of nude mice (Fig. 5A). Notably, tumor growth was largely reduced in regions containing STMN1-knockdown cells, but was maintained in regions containing shLuc control cells (Fig. 5B,C and D; n = 4), suggesting that STMN1 is crucial for tumor cell growth.

### STMN1 knockdown causes cell cycle redistribution

**Figure 6:**
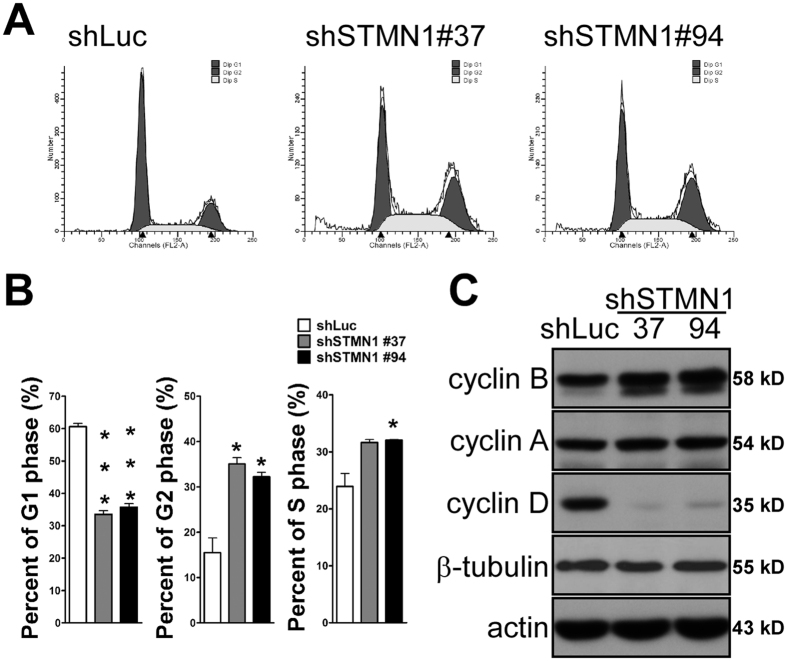
Cell cycle was redistributed in the knockdown of STMN1. (**A**) J7-shSTMN1 and control cells were grown to desired confluence. Cells were then trypsinized, fixed and stained with PI. DNA contents were analyzed by flow cytometry. Percent of cell cycle distributions were presented in (**B**). Error bars, s.e.m. (n = 3). (**C**) Immunoblots of J7-shSTMN1 and J7-shLuc cells. Gel electrophoresis was conducted under the same experimental conditions, and images of blots were cropped. Uncropped blot images are shown in Supplementary Fig. S3A.

To further investigate the molecular mechanism underlying STMN1-regulated cell growth, we analyzed the cell cycle distribution of parental and STMN1-knockdown J7 cells. Flow cytometry analyses indicated that the G2/M phase population was expanded in STMN1-knockdown cells, whereas the G1 phase population was reduced (Fig. 6A). Quantification of these results confirmed a 2-fold increase in the G2/M population in STMN1-knockdown cells compared with the Luc-knockdown control group (Fig. 6B). Moreover, STMN1 depletion was accompanied by increased levels of cyclin B and decreased levels of cyclin D, which modulate cell-cycle progression (Fig. 6C). These findings provide evidence for changes in cell-cycle progression upon suppression of STMN1 expression. Accordingly, we speculate that G2/M phase progression is retarded by knockdown of STMN1, leading to reduced cell proliferation.

### T_3_-mediated suppression of STMN1 contributes to cell growth arrest

**Figure 7:**
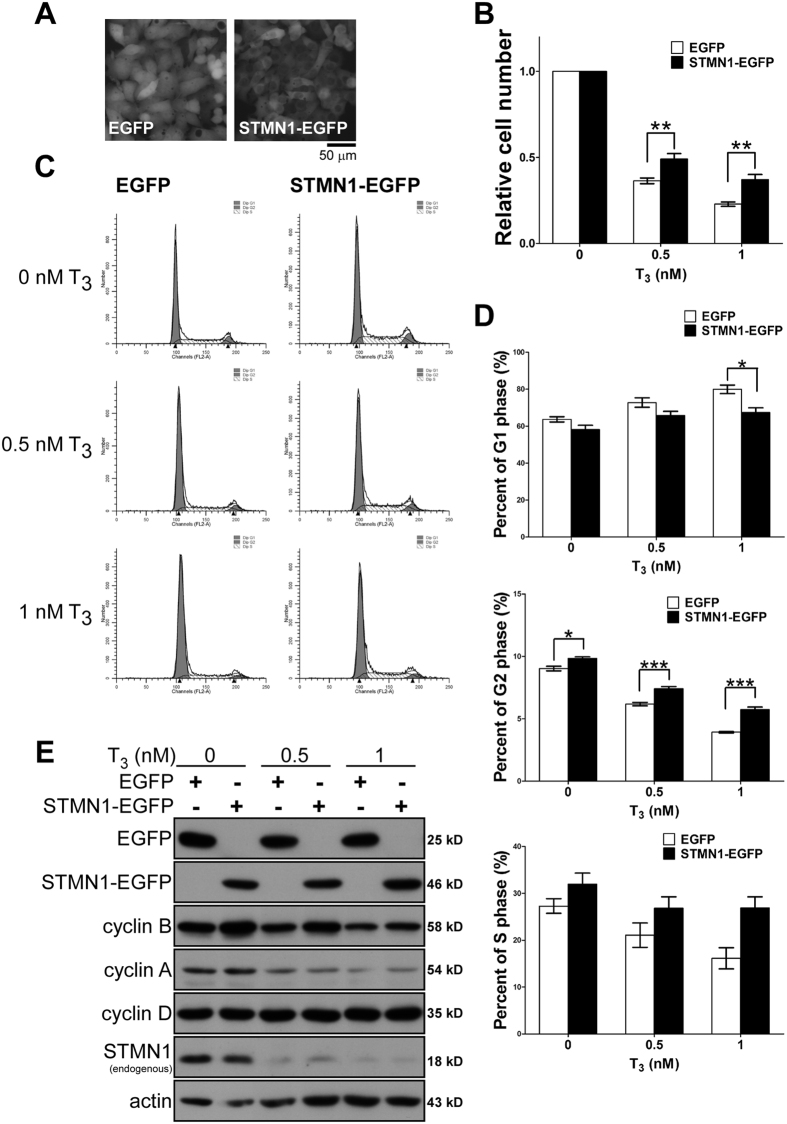
Overexpression of STMN1 attenuates T_3_-suppressed cell growth. (**A**) Fluorescent image of stable expressing STMN1-GFP fusion protein in HepG2-THRA cell. (**B**) Cell growth in the absent or present of T_3_ for 4 days were analyzed. The relative folds of T_3_-suppressed growth of each individual clone were normalized by 0 nM T_3_. (**C**) The STMN1 overexpressing and control cells were analyzed by flow cytometry. Percent of cell cycle distributions were presented in (**D**). (***P < 0.001; **P < 0.01; *P < 0.05 n = 3). Error bars, s.e.m. (**E**) Immunoblots of STMN1-EGFP and EGFP stable expressing cells in the absent or present of 4 days T_3_ treatment. Gel electrophoresis was conducted under the same experimental conditions, and images of blots were cropped. Uncropped blot images are shown in Supplementary Fig. S3B.

To further investigate the relevance of STMN1 in T_3_-mediated repression of cell growth, we restored STMN1 levels in HepG2-THRA cells by stably expressing STMN1-EGFP (Fig. 7A). T_3_ repressed cell growth through an increase in the G1 phase population and a reduction in the G2/S phase population (Fig. 7B–D); it also suppressed both cyclin B and cyclin A protein levels (Fig. 7E). In contrast, ectopic expression of STMN1 increased cell growth and expanded the G2 phase population in the presence of T_3_, and reduced the G1 phase population and caused accumulation of cyclin B (Fig. 7B–E). These data further confirm that STMN1 is involved in T_3_-induced cell growth arrest.

We conclude that STMN1, an essential protein for tumorigenic growth and cell-cycle progression, is inhibited by T_3_ at the transcriptional level, reflecting the negative correlation between the expression patterns of these proteins in clinical liver cancer specimens.

## Discussion

The present study confirmed that STMN1 is highly expressed in clinical HCC samples^39^. Our results further indicate that THR down-regulates STMN1 expression and show that its expression is negatively correlated with that of STMN1 in HCC, supporting the role of THR as a tumor suppressor. These results also establish THR as a new transcriptional regulator of *STMN1* gene expression. Notably, previous reports have indicated that both T_3_ treatment and STMN1 knockdown suppress cell growth^28^,^39^,^40^. However, the opposite changes in the cell cycle distribution caused by T_3_ treatment and STMN1 knockdown reveal differential regulation by these two interventions. We speculate that T_3_ treatment reduces the proliferation rate by causing accumulation of cells in G1 phase. By contrast, STMN1-knockdown cells lack normal microtubule turnover, leading to retardation in G2/M phase progression. This finding is in accord with previous reports^41^,^42^. However, G1 arrest has also been observed in STMN1-depleted gastric tumor cell lines^43^. These inconsistencies in cell cycle regulation reflect the different mechanisms of tumor proliferation in which STMN1 participates^44^. Our molecular evidence further revealed the cyclin B protein is elevated by STMN1 knockdown, whereas cyclin D is repressed. STMN1 rescue experiments designed clarify the role of STMN1 in T_3_-induced cell growth arrest confirmed that STMN1 partially restores T_3_ effects on cell growth, cell-cycle redistribution and expression of cyclins. These data also suggest that T_3_ acts at least in part through STMN1 to repress cell proliferation. However, the detailed molecular mechanism remains to be elucidated.

A previous investigation showed that, in normal liver subjected to partial hepatectomy, STMN1 expression is elevated in proliferating hepatocytes, but is silenced in resting hepatocytes, leading to the proposal that STMN1 expression is reversible and participates in tissue-specific proliferation–differentiation switching^45^. Other studies have revealed an oncogenic role of STMN1 based on its expression profiles in patients or effects of manipulating its expression on cellular tumorigenicity^39^,^40^,^46^. Silencing of STMN1 expression in the HCC cell line, HCCLM3, was shown to significantly reduce cell proliferation, adhesion and invasion, and trigger apoptosis^40^. Exogenous expression of E2F1 and the transcription factor DP-1 (TFDP1) has been shown to induce STMN1 mRNA expression^46^. Moreover, overexpression of STMN1 results in microtubule disassembly and is associated with formation of binucleated cells^39^. These data collectively indicate that THR-mediated suppression of STMN1 is required for normal liver maintenance. Impairment of this process may lead to constitutive STNM1 expression, and consequently, hepatic carcinogenesis.

T_3_ actions also extend to microtubules—the major cytoskeletal targets of STMN1—which are essential for internal vesicle transport and nervous system differentiation. Several studies have revealed that T_3_ regulates microtubule organization and is involved in microtubule-associated cell physiology^47^,^48^. Specifically, the rate of microtubule assembly *in vitro* is reduced in hypothyroid rats and restored upon administration of physiological levels of T_3_^47^. Notably, experiments using granulosa cells have shown that T_3_ dramatically reduces the effects of paclitaxel, a microtubule inhibitor used for disruption of mitotic microtubule assembly and cancer therapy^48^. Considered in this context, our observations suggest that T_3_ might regulate microtubule network assembly through repression of STMN1 expression.

## Conclusions

In summary, we have provided evidence that T_3_ suppresses proliferation via transcriptional regulation of STMN1 expression. Moreover, T_3_-regulated STMN1 expression may be associated with HCC malignancy.

## Materials and Methods

### Cell culture

Human HCC cell lines were cultured in Dulbecco’s modified Eagle’s medium supplemented with 10% (v/v) fetal bovine serum at 37 °C in a humidified 5% CO_2_ incubator. T_3_-depleted serum was prepared as described previously^49^. Briefly, an aliquot of serum (50 ml) was incubated with 2.2 g AG 1-X8 resin that had been washed three times with distilled, deionized water (15 min each), pelleted by brief centrifugation, and sterilized by autoclaving. T_3_ was depleted three times for at least 5 h each and filtered using a 0.22-μm filter.

### qRT-PCR

Total RNA was extracted using TRIzol (Invitrogen, 10296-028), as described previously^50^. cDNA was generated from 4 μg of purified total RNA using the Superscript^III^ kit for RT-PCR (Invitrogen). qRT-PCR was performed in a 15-μl reaction mixture containing 50 nM forward and reverse primers, 1× SYBR Green reaction mix (Applied Biosystems, 4309155) and 48 μg template, according to a previously described protocol^50^. The sequences of forward (q-STMN1-F) and reverse (q-STMN1-R) primers used were 5′-GTG GTC AGG CGG CTC GGA CTG-3′ and 5′-CTC TCG TTT CTC AGC CAG CTG C-3′, respectively.

### Northern blotting

Cellular RNA was denatured by heating at 75 °C for 15 min and then chilled on ice for 5 min. Denatured RNAs were resolved on a 1.2% agarose gel and transferred to a nylon membrane (Amersham Bioscience, UK) overnight. The membrane was cross-linked and blocked by prehybridization in the presence of 250 μg/ml single-strand DNA for at least 8 h. α-^32^P-dCTP* was incorporated into the STMN1 probe by PCR. The sequences of forward (STMN1-F) and reverse (STMN1-R) primers used were 5′-ATG GCT TCT TCT GAT ATC-3′ and 5′-TTA GTC AGC TTC AGT CTC-3′, respectively. The membrane was incubated with radiolabeled probe at 42 °C for 18 h. After a brief wash, the remaining isotope signal was detected with X-ray film (Amersham Bioscience).

### Immunohistochemical staining

Tumor tissue microarrays were purchased from Taiwan Liver Cancer Network (TCLN) and constructed to contain 115 normal liver tissues and 115 hepatocellular carcinoma samples. The staining score (Quick score) was calculated using the following formula:


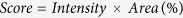


Staining intensity was rate on a 0 to 3 scale, where 0 is negative, 1 is weak, 2 is moderate and 3 is strong; the area percentage reflects the degree of positive staining^51^. This study was approved by the Institutional Review Board of Chang Gung Medical Center Human Ethics Committee (IRB No: 99-3588B). The study subjects provided written informed consent, and all methods were carried out in accordance with the approved guidelines.

### SDS-PAGE and immunoblot analysis

Total cell extracts were purified as described previously^50^. Equal amounts of protein were resolved by sodium dodecyl sulfate-polyacrylamide gel electrophoresis (SDS-PAGE) and transferred to a PVDF (polyvinylidene difluoride) membrane using a semi-dry transfer system. The membrane was blocked by incubating with 5% non-fat milk dissolved in phosphate-buffered saline containing 0.1% Tween-20 (PBST) for 1 h, and then was incubated with the appropriately diluted primary antibody at 4 °C overnight. After washing with PBST, the membrane was incubated with the appropriate secondary antibody for 2 h at room temperature. Immune complexes were developed by chemiluminescence using an enhanced chemiluminescence (ECL) detection kit (Amersham, RPN2232)^50^.

### Antibodies and reagents

The following antibodies and reagents were used: rabbit polyclonal anti-STMN1 (Calbiochem, 569391), rabbit polyclonal anti-THRA (GeneTex, GTX25621), mouse monoclonal anti-THRB (Santa Cruz, SC-737), mouse monoclonal anti-β-actin (Chemicon, MAB1501R), rabbit monoclonal anti-cyclin B1 (Abcam, ab32053), rabbit monoclonal anti-cyclin A (Abcam, ab185619), rabbit monoclonal anti-cyclin D1 (Epitomics, 4202-1), mouse monoclonal anti-β-tubulin (Chemicon, MAB3408), AG 1-X8 resin (Bio-Rad, 140-1451), T_3_ (Sigma-Aldrich, T2752), and cycloheximide (Sigma-Aldrich, C7698). Mouse monoclonal anti-THR (C4) was a gift from Sheue-yann Cheng (National Cancer Institute).

### Plasmid construction

Constructs containing potential THR-regulated sites of the *STMN1* promoter were generated by PCR-amplification of an upstream region of the *STMN1* gene corresponding to nucleotides −2891 to +1 (where +1 corresponds to the AUG initiation site) and serially deleted fragments, and subcloning them into pGL3-Luc or pA3tk-Luc plasmids. The primer sequences and restriction sites used were as follows:

STMN1(−2891)-*Kpn*I-F: 5′-AAATAAGGTACCTCAAAGCAGGTGTCTTGGTG-3′, STMN1(−2241)-*Kpn*I-F: 5′-AAATAAGGTACCGTCTTAGGCACCCATGTGGG-3′, STMN1(−1943)-*Kpn*I-F: 5′-AAATAAGGTACCCATTGTCCTCCTGCCCTCCG-3′, STMN1(−1191)-*Kpn*I-F: 5′-AAATAAGGTACCAGCTTGGGTGGCGGCAGGTT-3′, STMN1(−701)-*Kpn*I-F: 5′-AAATAAGGTACCGGTACTAGCTGGCGTCTACA-3′, STMN1(−450)-*Kpn*I-F: 5′-AAATAAGGTACCCAATGAGTTGTAGGCAGTAT-3′, STMN1(−250)-*Kpn*I-F: 5′-AAATAAGGTACCATATTCAGGTCATATTTCCC-3′, STMN1(−100)-*Kpn*I-F: 5′-ATAAGGTACCAAAGAAAGTGATTGCATGTTTT-3′, STMN1(+1)-*Nhe*I-R: 5′-ACTATCGCTAGCTGGTGAATAGAAGACAAGCG-3′, STMN1(−101)-*Nhe*I-R: 5′-ACTATCGCTAGCGCCTTTCTATATGTCAT-3′.

The STMN1-EGFP fusion plasmid was generated by first PCR-amplifying STMN1 fragments and enhanced green fluorescent protein (EGFP) using primer pairs with the indicated sequences and incorporated restriction sites:

STMN1-*Bam*HI-F, 5′-AAT CGG ATC CAT GGC TTC TTC TGA TAT CCA GG-3′ (forward) and STMN1-EGFP-R, 5′-CCC TTG CTC ACC ATG TCA GCT TCA GTC TCG TCA GCA G-3′ (reverse); and EGFP-STMN1-F, 5′-CTG CTG ACG AGA CTG AAG CTG ACA TGG TGA GCA AGG G-3′ (forward) and EGFP-*Hin*dIII-R, 5′-GAT CAA GCT TTT ACT TGT ACA GCT CGT CCA TG-3′ (reverse).

STMN1-EGFP was conjugated by mixing the two PCR products and performing a further extension step, followed by subcloning into the pcDNA3.1/Hygro plasmid.

### Promoter-reporter and ChIP assays

HepG2-THRA cells were transfected overnight with *STMN1* promoter vectors using the TurboFect reagent (Thermo Fisher Scientific, R0531). Transfected cells were incubated in the presence or absence of T_3_ for an additional 48 h. After treatment, cells were lysed for the detection of firefly luciferase activity (Promega, E1960).

ChIP assays were performed as described previously^52^. Briefly, HepG2-THRA cells were fixed to cross-link DNA-protein complexes, and sonicated on ice to obtain bulk DNA fragments with a size range of ~200–400 bp. THR- or RXRA-binding DNA was immunoprecipitated with the corresponding antibody, and complex crosslinking was reversed by heating at 65 °C overnight. Targeted elements were detected by amplifying purified DNA fragments using primer pairs specific for the STMN1 TRE. The glyceraldehyde 3-phosphate dehydrogenase (GAPDH) promoter region was used as a negative control. The following primer pairs were employed to detect specific promoter regions: ChIP-STMN1-F, 5′-AAA GAA AGT GAT TGC ATG TTT TTG AAA ATC-3′ (STMN1 forward) and ChIP-STMN1-R, 5′-TGG TGA ATA GAA GAC AAG CGA CAG-3′ (STMN1 reverse); ChIP-GAPDH-F, 5′-CAA GGC TGA GAA CGG GAA GC-3’ (GAPDH forward) and ChIP-GAPDH-R, 5′-AGG GGG CAG AGA TGA TGA CC-3′ (GAPDH reverse).

### Knockdown and overexpression of STMN1

For knockdown of endogenous STMN1, J7 cells were infected with lentivirus expressing shRNA targeting STMN1. The pLKO.1 shLuc (control) and pLKO.1 shSTMN1 expression vectors were obtained from the National RNA Interference Core Facility (Institute of Molecular Biology, Academia Sinica, Taipei, Taiwan). Clones TRCN0000072243 and TRCN0000160037 correspond to shLuc and shSTMN1, respectively. For overexpression of STMN1, HepG2-THRA cells were transfected with an STMN1-EGFP expression plasmid. EGFP-positive cells were further enriched by flow cytometry.

### Proliferation and colony-formation assays

For analysis of cell proliferation, 1 × 10^5^ cells were seeded in wells of a 6-well plate. Viable cells were trypsinized and counted on the indicated days. Colony-formation activity was analyzed by seeding 2 × 10^3^ cells in 6-well plates for 14 d. After incubating for 2 weeks, colonies were fixed and stained with crystal violet. Colony numbers and occupied areas were scanned and measured using ImageJ software.

### Murine tumor-progression model

SCID mice were used to assess the *in vivo* growth potential of J7-shSTMN1-derived tumors. Briefly, J7-shLuc and J7-shSTMN1 cells (1 × 10^6^) were suspended in 150 μl of PBS and injected into the dorsal skin of mice (left, shLuc; right, shSTMN1). After 2 weeks, visible tumors were measured every 2–3 d. Tumor volume was calculated using the following formula:


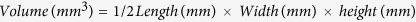


Xenograft tumors were subsequently dissected and individually weighed. Animal care procedures were in accordance with the Chang-Gung Institutional Animal Care and Use Committee Guide for the Care and Use of Laboratory Animals (CGU08-05), and all methods were approved by the Chang-Gung Institutional Animal Care and Use Committee.

### Flow cytometry

For cell-cycle analysis, cells were harvested by trypsin digestion and fixed in 75% ethanol for at least 1 h at −20 °C. Cells were then treated with 0.5% Triton X-100 and 0.05% RNase A for 1 h at 37 °C. Thereafter, nuclear DNA was incubated with 50 μg/ml propidium iodide stain for 20 min at 4 °C, and cells were analyzed on a FACSCalibur flow cytometer (Becton Dickinson Immunocytometry Systems, CA, USA).

### Statistical analysis

Values are expressed as means ± s.e.m. Statistical analyses of data were performed using Student’s t-test or one-way analysis of variance (ANOVA), as appropriate. A *P*- value < 0.05 was considered significant.

## Additional Information

**How to cite this article**: Tseng, Y.-H. *et al*. Thyroid hormone suppresses expression of stathmin and associated tumor growth in hepatocellular carcinoma. *Sci. Rep.* **6**, 38756; doi: 10.1038/srep38756 (2016).

**Publisher's note:** Springer Nature remains neutral with regard to jurisdictional claims in published maps and institutional affiliations.

## Supplementary Material

Supplementary Figure
