# Supplementary material for: Thyroid hormone suppresses expression of stathmin and associated tumor growth in hepatocellular carcinoma
Source: Sci Rep. 2016 Dec 9;6:38756. doi: 10.1038/srep38756 (PMC5146664; doi:10.1038/srep38756)

Thyroid hormone suppresses expression of stathmin and associated tumor growth in hepatocellular carcinoma

Yi-Hsin Tseng[1], Ya-Hui Huang[2], Tzu-Kang Lin[3], Sheng-Ming Wu[1], Hsiang-Cheng Chi[1], Chung-Ying Tsai[1], Ming-Ming Tsai[4], Yang-Hsiang Lin[1], Wei-Chun Chang[1], Ya-Ting Chang[5], Wei-Jan Chen[6], and Kwang-Huei Lin[1,2]

1. Graduate Institute of Biomedical Sciences, College of Medicine, Chang Gung University, Taoyuan 333, Taiwan, Republic of China.
2. Liver Research Center, Chang Gung Memorial Hospital, Linko, Taoyuan 333, Taiwan, Republic of China.
3. Division of Neurosurgery, Chang Gung Memorial Hospital-Linkou & Chang Gung University, Taoyuan 333, Taiwan, Republic of China.
4. Department of Nursing, Chang-Gung University of Science and Technology, Taoyuan 333, Taiwan, Republic of China.
5. Molecular Medicine Research Center, Chang Gung University, Taoyuan 333, Taiwan, Republic of China.
6. First Cardiovascular Division, Chang Gung Memorial Hospital, Taoyuan 333, Taiwan, Republic of China.

Corresponding author:

Dr. Kwang-Huei Lin

Graduate Institute of Biomedical Sciences, Chang Gung University, 259 Wen-Hwa 1st Road, Taoyuan 333, Taiwan, Republic of China

Phone: +886-3-2118263

Fax: +886-3-2118263

E-mail: khlin@mail.cgu.edu.tw

Supplementary Figure S1. T_3 represses STMN1 expression in HepG2-THRA cells.

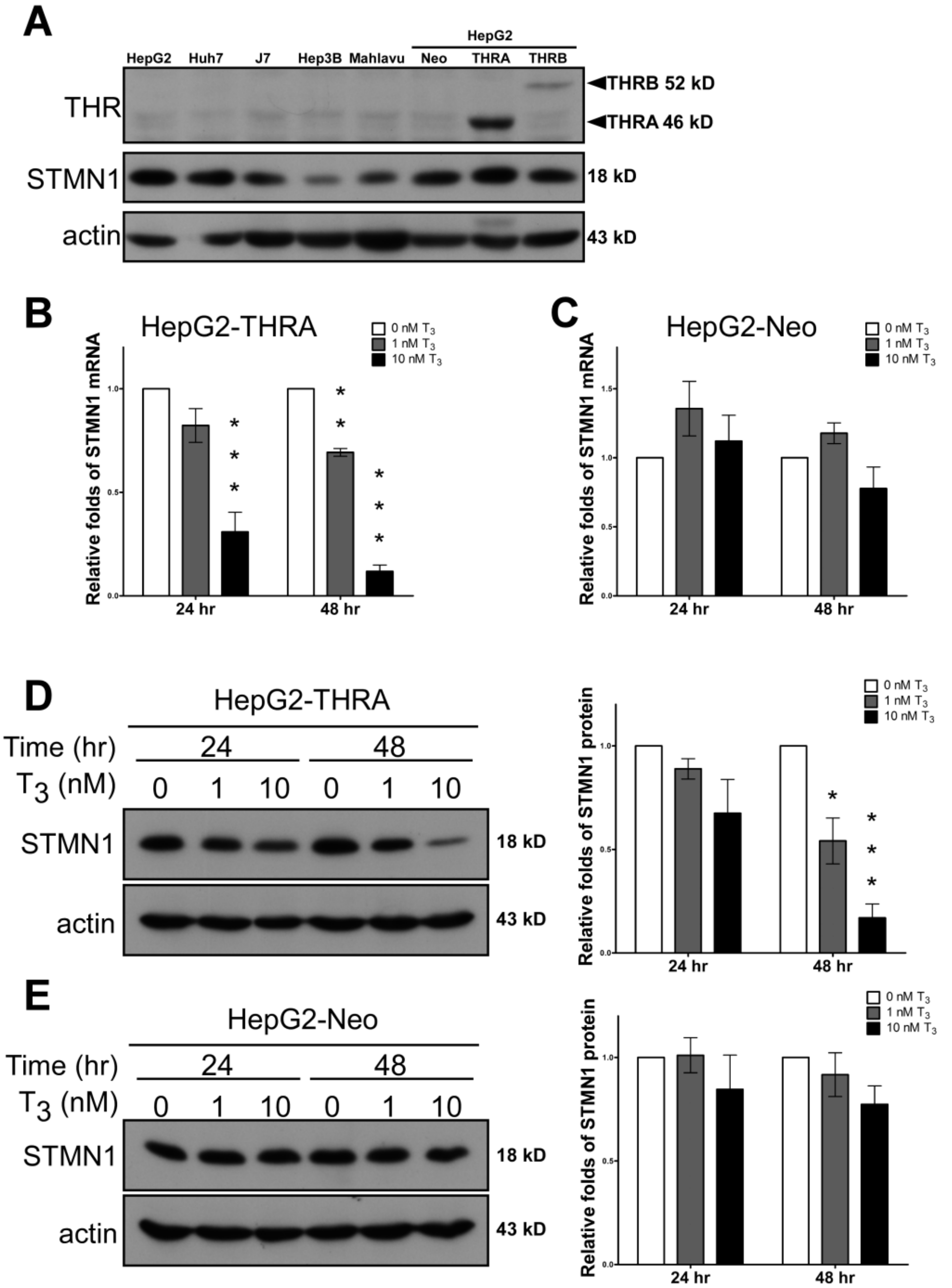

(**A**) THR and STMN1 protein expression levels in HCC and THR overexpressing cell lines. (**B** and **C**) qRT-PCR of STMN1 mRNA expression levels in HepG2-THRA and HepG2-Neo control cells (**$P<0.01$, ***$P<0.001$, n=3). (**D** and **E**) Immunoblots of HepG2-THRA and HepG2-Neo cell lysates (*$P<0.05$, ***$P<0.001$, n=3). Error bars, s.e.m. Electrophoretic gels and blots in each experiment were conducted under the same experimental conditions, and images were cropped.

Supplementary Figure S2. Basal Luc activities of promoter assay in Figure 3A.

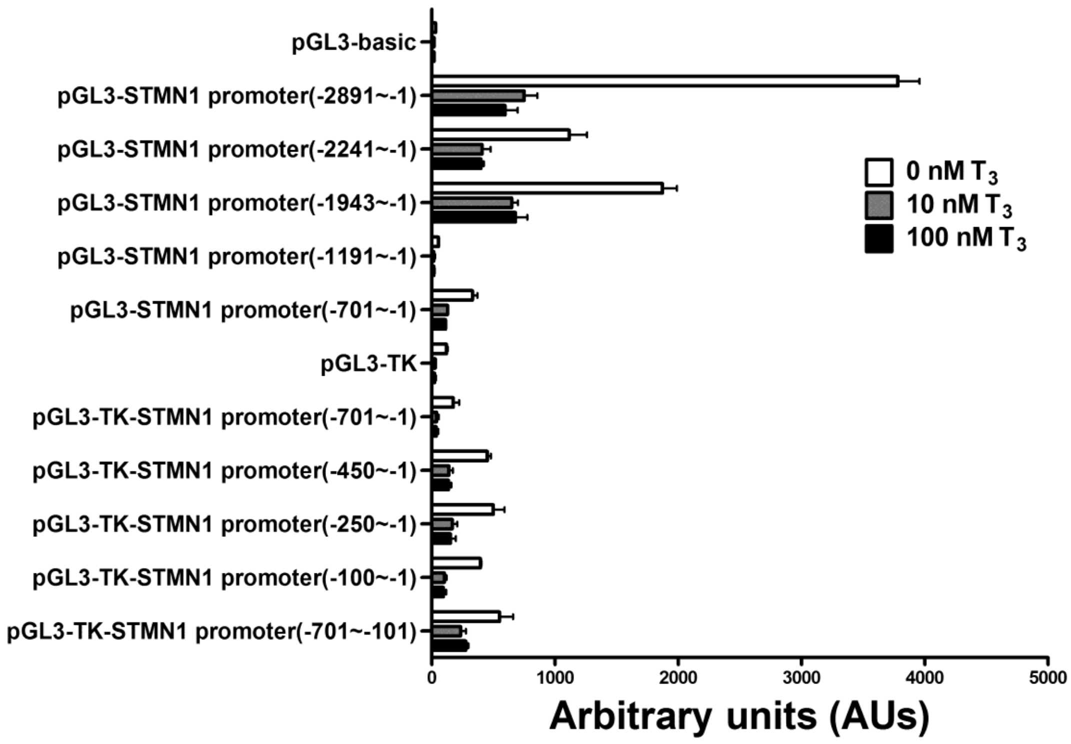

Supplementary Figure S3. Uncropped blot images of Figure 6C and Figure 7E.

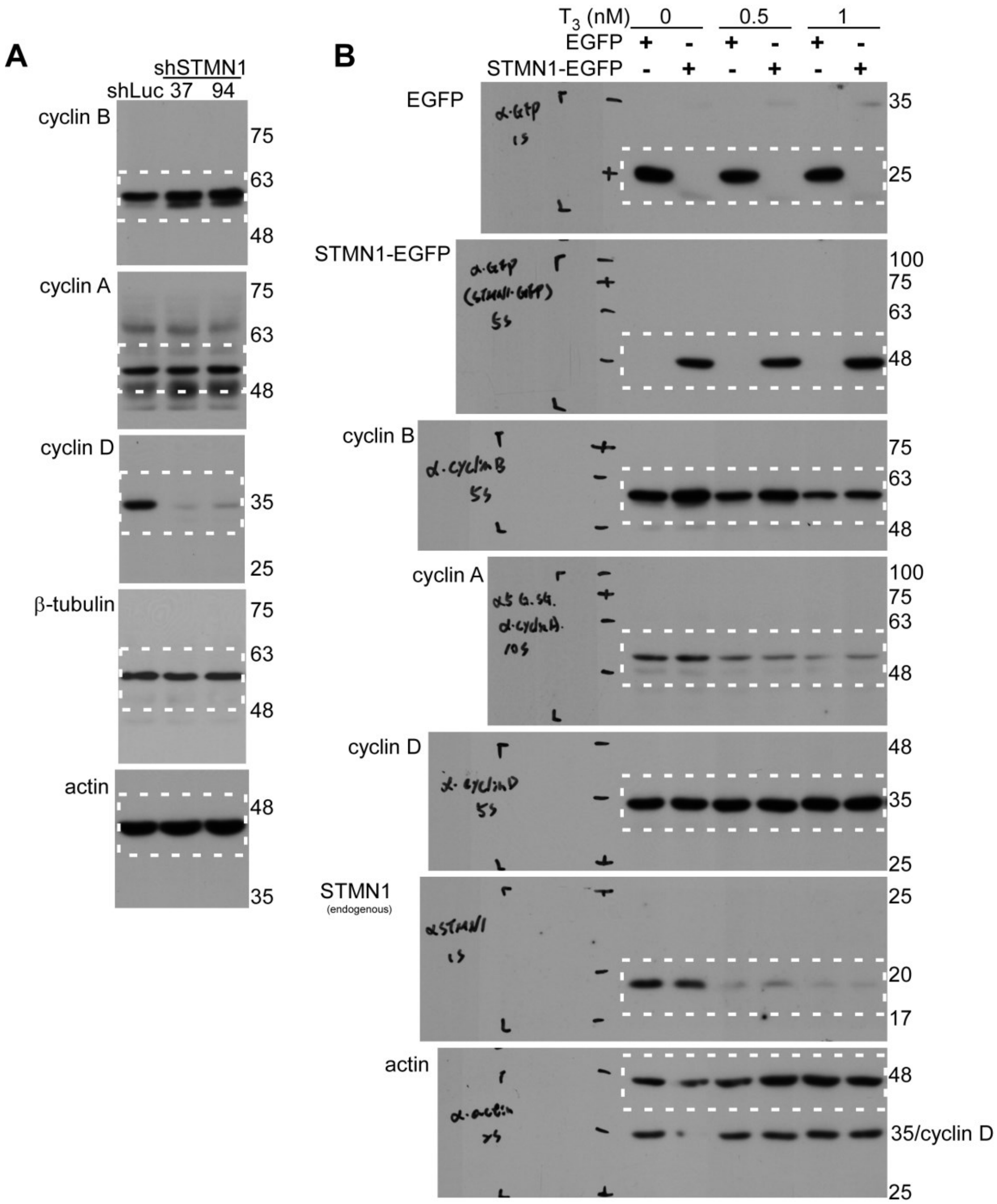

Supplement: Supplementary Figure [file srep38756-s1.pdf]
